# Model for Predicting Serious Hematological Adverse Events in Individuals With Ovarian Cancer Receiving Poly (Adenosine Diphosphate Ribose) Polymerase Inhibitor Treatment: Prospective Cohort Study

**DOI:** 10.2196/72994

**Published:** 2025-11-12

**Authors:** Xiaotong Lian, Yu Lei

**Affiliations:** 1 Guangxi Medical University Nanning China; 2 Guangxi Medical University Affiliated Tumor Hospital Nanning China

**Keywords:** poly (adenosine diphosphate ribose) polymerase inhibitors, PARPis, hematological adverse event, ovarian cancer, risk prediction model

## Abstract

**Background:**

Predicting serious hematological adverse events (SHAEs) from poly (adenosine diphosphate ribose) polymerase inhibitors (PARPis) would allow us to prioritize patients with ovarian cancer at higher risk for more intensive care, ultimately lowering morbidity and preventing them from premature termination of medication.

**Objective:**

This study aimed to explore the risk factors for SHAEs in patients with ovarian cancer receiving PARPi treatment and develop a risk prediction model for such events.

**Methods:**

Prospective clinical data were collected on patients with ovarian cancer who received PARPi treatment at the Guangxi Medical University Affiliated Tumor Hospital from December 2018 to August 2024. They were divided into a SHAE group and a no-SHAE group based on the occurrence of SHAEs. Variable differences were screened using the chi-square test or Fisher exact test. Multivariate logistic regression was used to determine independent factors influencing SHAEs in patients with ovarian cancer. A predictive model for serious blood-related complications in ovarian cancer treatment was developed from identified independent risk factors using the R software. The model’s clinical utility was assessed through decision curve analysis (net benefit), calibration (calibration curve), and discrimination (receiver operating characteristic curve).

**Results:**

A total of 70 patients with ovarian cancer receiving PARPi treatment were included in this study. Of these 70 patients, 16 (23%) experienced SHAEs, with decreases in red blood cell (RBC) count and hemoglobin levels being the most common. Multiple logistic regression analysis identified 4 independent predictors of PARPi-associated SHAEs in patients with ovarian cancer: lymph node metastasis (odds ratio [OR] 6.733, 95% CI 1.197-37.873; *P*=.03), creatinine clearance rate of ≤60 mL per minute (OR 23.722, 95% CI 3.121-180.303; *P*=.002), RBC count of ≤3.3×10^12^ per liter (OR 4.847, 95% CI 1.020-23.041; *P*=.047), and combination therapy with vascular endothelial growth factor inhibitors (OR 6.749, 95% CI 1.313-34.689; *P*=.02). The internal validation yielded an area under the curve of 0.874 (95% CI 0.793-0.955), indicating moderate clinical utility and accuracy for the risk prediction model incorporating these predictors.

**Conclusions:**

Lymph node metastasis, creatinine clearance rate of ≤60 mL per minute, RBC count of ≤3.3×10^12^ per liter, and combination therapy with vascular endothelial growth factor inhibitors are independent risk factors for PARPi SHAEs in patients with ovarian cancer. The risk prediction model established based on these factors demonstrated moderate predictive value.

## Introduction

### Background

Ovarian cancer is the most aggressive gynecologic malignancy. Most patients with ovarian cancer are diagnosed in later stages due to unclear early symptoms and its anatomical location deep in the pelvis. Even after surgery, it is still prone to recurrence and metastasis, which has a serious impact on women’s lives and health. Approximately 70% of patients still experience recurrence and develop chemotherapy resistance within 3 years after initial treatment despite showing sensitivity in the early stages of chemotherapy [1,2]. Given the frequent and rapid development of chemotherapy resistance in ovarian cancer, there is an urgent clinical need to identify new treatment options. High-level medical evidence shows that poly (adenosine diphosphate ribose) polymerase inhibitors (PARPis) can interfere with the base excision repair pathway, block the DNA single-strand break repair of tumor cells, form a “synthetic lethal” effect in tumor tissues that cannot undergo DNA double-strand repair due to homologous recombination repair deficiency (HRRD), promote tumor cell apoptosis, effectively prolong the platinum-free interval of patients with ovarian cancer, and provide a new treatment option for patients who cannot tolerate chemotherapy in the late stage [3]. Germline mutations in the *BRCA1* and *BRCA2* genes, which are ovarian cancer susceptibility genes, are common causes of HRRD. Therapeutic benefits of PARPis have been observed in patients with cancer carrying germline mutations in the *BRCA1* and *BRCA2* genes, particularly for ovarian cancer cases [4]. In terms of safety, serious adverse reactions to PARPis are mainly manifested in hematological toxicity and are most likely to lead to drug reduction, interruption, or even discontinuation, seriously hindering the normal treatment process of patients with ovarian cancer and causing adverse effects on their long-term prognosis [5,6]. Therefore, identifying serious hematological adverse events (SHAEs) related to PARPis in advance is particularly important in clinical diagnosis and treatment practice.

### Objectives

This study aimed to explore the risk factors for SHAEs in patients with ovarian cancer receiving PARPi treatment and develop a risk prediction model to guide personalized treatment and care for patients with ovarian cancer to achieve the best survival benefits.

## Methods

### Study Design

This open-label, prospective cohort study was designed to evaluate the incidence of SHAEs in patients with ovarian cancer receiving PARPi treatment in routine clinical situations and determine the variables linked to the emergence of these reactions. This study included patients aged ≥18 years with histologically proven ovarian, fallopian tube, or primary peritoneal cancer who received care at the Guangxi Medical University Affiliated Tumor Hospital from December 2018 to August 2024. All participants (or their legal representatives) provided informed consent, and all had been treated with PARPis for a minimum duration of 6 weeks in any treatment line. The exclusion criteria were the participants’ or the responsible family members’ withdrawal of permission to take part in the research at any time or PARPi discontinuation due to disease progression or death.

### Study Procedures and Assessments

Participants were followed up on on-site or contacted via telephone calls at least once a month to inquire about the incidence of any untoward effect from PARPis from initiation until they completed their treatment, were lost to follow-up, or died between December 2018 and August 2024. Patients were evaluated for treatment-related hematological indicators and imaging of tumor lesions every 2 to 3 treatment cycles. Any hematological adverse event (including time of occurrence, severity, and prognosis) was also included in the data recording. When to stop treatment in case of an adverse event was decided by the attending physician.

The safety data were collected using the National Cancer Institute Common Terminology Criteria for Adverse Events version 5.0. Depending on whether there were SHAEs during the follow-up period, the patients were split into 2 groups: the SHAE group and the no-SHAE group. SHAEs are defined as hematological adverse events of a grade of ≥3, including thrombocytopenia, leucopenia, neutropenia, erythropenia, decreased hemoglobin, anemia, myelodysplastic syndrome, and acute myeloid leukemias. If none of the aforementioned SHAEs occurred, patients were included in the no-SHAE group. In addition, when a single report contained multiple adverse events, if any of the aforementioned serious adverse events occurred, patients were included in the SHAE group.

The outcomes of each patient were assessed by combining the Response Evaluation Criteria in Solid Tumors version 1.1 and the Gynecologic Cancer Intergroup criteria, including disease control rate (DCR), partial response, stable disease, and progressive disease.

### Establishment of the Nomogram Model

General clinical characteristics of patients before PARPi treatment, including age, BMI, marital status, pathology type, previous antitumor therapies, lymph node metastasis, distant metastasis, and number of distant metastases, were collected. Univariate analyses were conducted for each parameter using the chi-square test or Fisher exact test, as appropriate. Variables with a significance level of *P*<.20 in the univariate analysis were included in the subsequent multivariate logistic regression analysis using a backward elimination approach (removal criterion: *P*>.05 based on the Wald statistic) to identify independent risk factors for SHAEs during PARPi treatment in patients with ovarian cancer. For all binary categorical variables (eg, lymph node metastasis), the reference category was explicitly set to the negative group (coded as 0) to ensure clinically intuitive interpretation of odds ratios. Statistical analyses were conducted using SPSS (version 23.0; IBM Corp). Subsequently, the final multivariate model was imported into the R software (version 4.2.2; R Foundation for Statistical Computing), and the *rms* software package was used to construct a nomogram for predicting the risk of SHAEs.

### Validation of the Nomogram Model

By charting the receiver operating characteristic (ROC) curve and computing the area under the curve (AUC), the differentiation of the model was assessed. To confirm the model’s consistency, a calibration curve was created by contrasting the projected and actual probabilities of SHAEs. The clinical usefulness of the prediction model was assessed using decision curve analysis. To quantify the net benefit at various threshold probabilities, decision curve analysis compares the model’s performance to 2 reference lines: one that assumes that all patients experience SHAEs and receive the intervention (which represents the highest clinical cost) and one that assumes that no patients receive the intervention (which represents no clinical benefit).

### Statistical Analysis

Data were analyzed using SPSS (version 23.0). Categorical variables are presented as frequencies and percentages and were compared using the chi-square test or Fisher exact test, as appropriate. Cases categorized as “unknown” were excluded from the analysis to ensure validity and minimize potential bias. In the tables, variables with missing data are marked with a footnote symbol, indicating that these cases were excluded. For variables with a high proportion of missing values (≥50%), no statistical comparisons were conducted, and *P* values are denoted using an em dash (ie, not applicable).

Survival outcomes were assessed using the Kaplan-Meier method. To evaluate the association between the occurrence of serious hematological toxicity and clinical efficacy, a comparative analysis of progression-free survival (PFS) was conducted between participants who experienced any hematological adverse events of a grade of ≥3 during the treatment period and those who did not. The 2 groups were compared using the log rank test. Time-to-event end points specific to hematological adverse event occurrence were analyzed using survival analysis. The primary end point was time to first onset of hematological adverse events of a grade of ≥3, and secondary end points included time to first onset of any-grade hematological adverse events. The event was defined as the first documented occurrence of a hematological adverse event (including anemia, neutropenia, and thrombocytopenia) meeting the specified severity grade. Data from patients who did not experience an event were censored at the date of their last follow-up. The cumulative probability of each end point was estimated using the Kaplan-Meier method and visualized via Kaplan-Meier curves.

The associated factors influencing SHAEs following PARPi medication were examined using a multivariate logistic regression model, and a prediction model was created. The ROC curve was used to verify the model’s prediction ability, and the AUC was calculated. The degree to which the prediction model fit the data was assessed using the Hosmer-Lemeshow test. The difference was considered statistically significant if *P*<.05.

### Ethical Considerations

The protocol for this study was approved by the Ethics Review Board of Guangxi Medical University Affiliated Tumor Hospital (KY2023772). All procedures were conducted in accordance with the ethical standards of the Declaration of Helsinki and local regulations. Written or verbal informed consent was obtained from all participants (or their legal guardians) before their inclusion in the study.

To ensure participant privacy and confidentiality, all personal information was handled with strict adherence to our ethical protocols. Data collection forms were anonymized and stored separately from any personal identifiers. Access to the original dataset was restricted to authorized research personnel only. Throughout the data analysis and publication process, all participant data were de-identified to prevent any possibility of tracing back to individuals. Specifically, all direct personal identifiers (such as names, patient ID numbers, and detailed contact information) were removed from the research dataset.

## Results

### Overview

**Figure 1:**
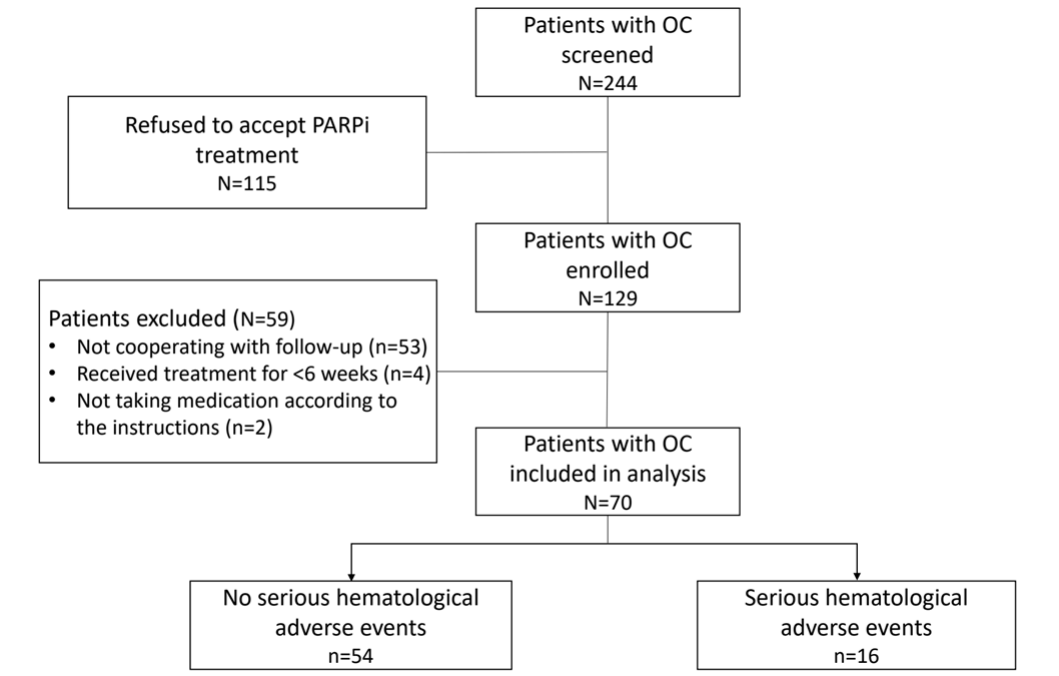
Flow diagram of this study. OC: ovarian cancer; PARPi: poly (adenosine diphosphate ribose) polymerase inhibitor.

Figure 1 shows the study sample recruitment. Of the 244 patients with ovarian cancer surveyed at baseline, 115 (47.1%) were not eligible for follow-up due to refusal. Of the 129 participants receiving PARPi treatment, we excluded 59 (45.7%) for the following reasons: 53 (90%) did not return for any follow-up visit and could not be reached for the entire study duration, 4 (7%) received treatment for <6 weeks, and 2 (3%) did not take their medications according to the instructions. As a result, the total sample size for the analysis was 70 patients.

The follow-up period lasted a median of 12 (IQR 7-24) months.

Of the 70 patients with ovarian cancer treated with PARPis, 16 (23%) experienced hematological adverse events of a grade of ≥3 and were classified as the SHAE group, and the 54 (77%) remaining individuals were categorized as the no-SHAE group. After the clinical characteristics of the individuals in the 2 groups were analyzed, the findings (Multimedia Appendix 1) demonstrated that there were statistically significant differences in creatinine clearance rate (Ccr), red blood cell (RBC) count, and response to 6-week treatment among the groups (*P*<.05).

### Assessment of Effectiveness and Prognosis

**Figure 2:**
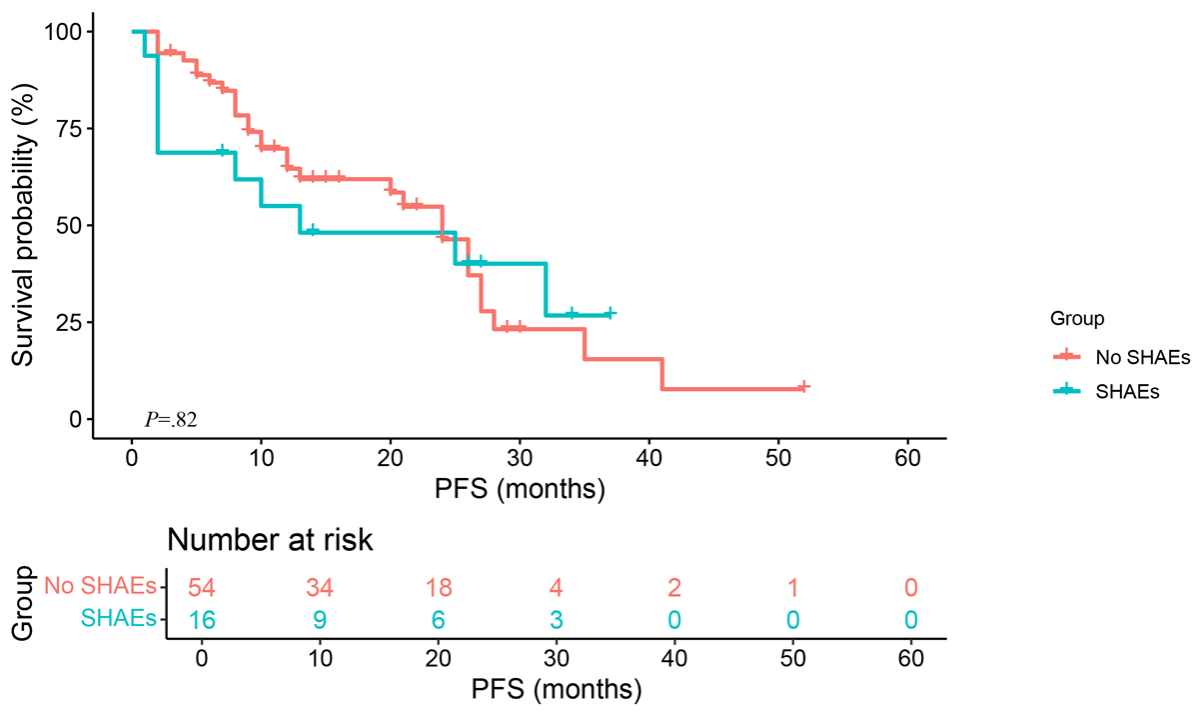
Progression-free survival (PFS) compared between participants who experienced serious hematological adverse events (SHAEs) and those who did not.

Treatment response was assessed in all study patients through combined application of the Response Evaluation Criteria in Solid Tumors version 1.1 and the Gynecologic Cancer Intergroup criteria. The results showed that progressive disease developed in 56% (39/70) of the patients, stable disease developed in 44% (31/70) of the patients, and partial response developed in 0% of the patients. A DCR was present in 44% (31/70) of the entire population. A significant difference was not found in PFS between the SHAE group and the no-SHAE group according to the results of a Kaplan-Meier survival analysis conducted for PFS in the 2 groups (*P*=.82), and the median PFS was 13.0 (IQR 0.0-38.4) months and 24.0 (IQR 19.2-28.8) months, respectively (Figure 2).

Further analysis of the relationship between SHAEs in patients with ovarian cancer and the efficacy of PARPis did not reveal any association between the early occurrence of SHAEs related to PARPi hematological toxicity and antitumor efficacy (Table 1).

**Table 1:** Disease control rates (DCRs) and absolute frequencies by adverse event experiences.

Time before initial event (mo)			Total, n		Disease control, n (%)		*P* value
**Adverse events**							.43
	≤1	8		5 (62.5)			
	>1	32		13 (40.6)			
**Serious hematological adverse events**							.30
	≤1	5		3 (60.0)			
	>1	11		3 (27.3)			

### Presentation of Hematological Adverse Events in Patients

**Figure 3:**
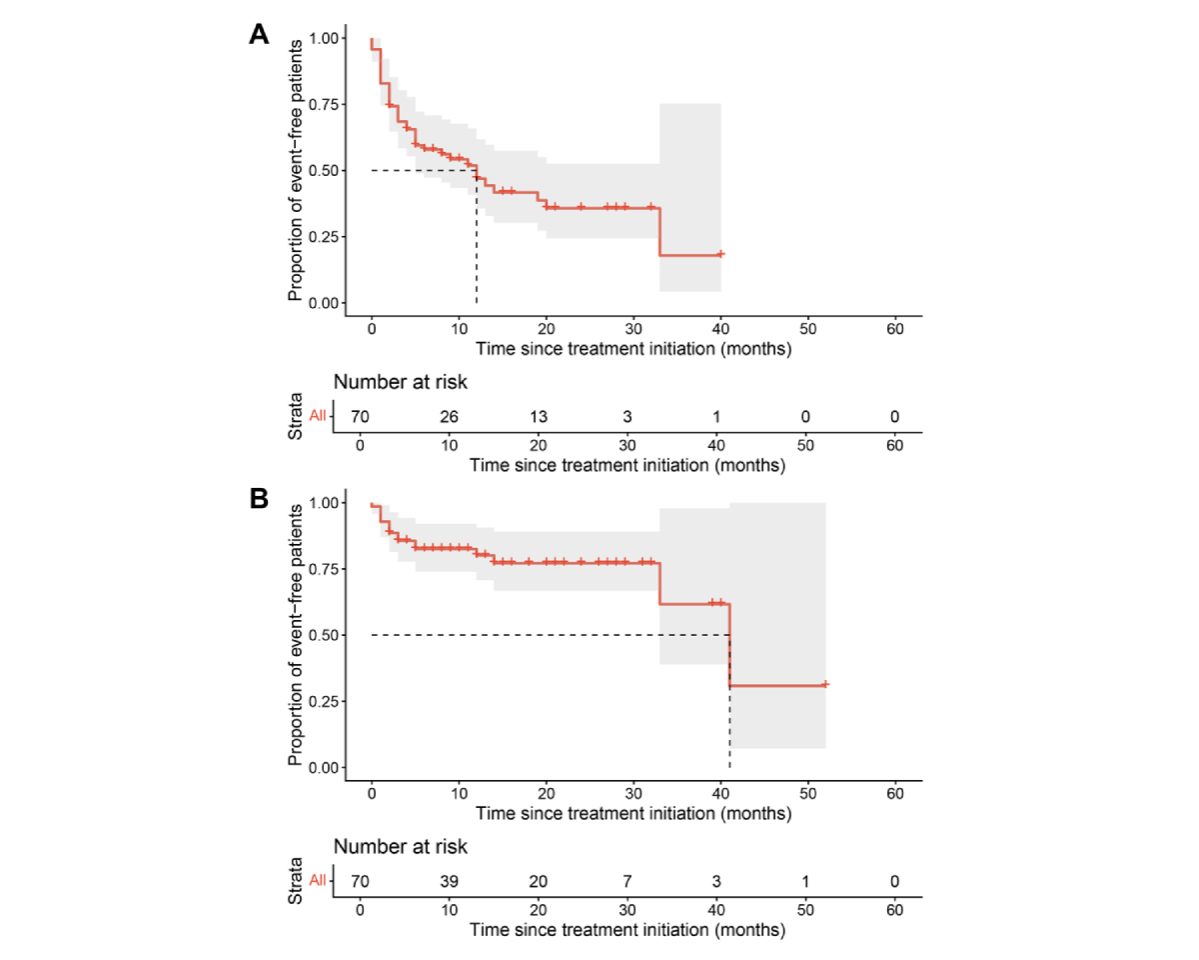
Time to first onset of hematological adverse events―(A) time to all-grade hematological adverse events; (B) time to (serious) hematological adverse events of a grade of ≥3.

The spectrum and classification of hematological adverse events are shown in Table 2. Erythropenia and decreased hemoglobin showed the highest incidence regardless of severity. Figure 3 illustrates the onset time of hematological adverse events during PARPi treatment. All-grade hematological adverse events had a median onset time of 12.0 (IQR 6.5-17.468) months, whereas hematological adverse events of a grade of ≥3 manifested later (median onset time 41.0, IQR 29.3-52.7 months).

**Table 2:** Incidence and types of hematological adverse events (N=70).

	Grade <3, n (%)	Grade ≥3, n (%)	Total, n (%)
Any hematological adverse event	23 (33)	17 (24)	40 (57)
Thrombocytopenia	5 (7)	10 (14)	15 (21)
Leucopenia	12 (17)	8 (11)	20 (29)
Neutropenia	7 (10)	8 (11)	15 (21)
Erythropenia	16 (23)	12 (17)	28 (40)
Decreased hemoglobin	15 (21)	13 (19)	28 (40)
Anemia	6 (9)	11 (16)	17 (24)
Myelodysplastic syndrome	0 (0)	0 (0)	0 (0)
Acute myeloid leukemias	0 (0)	0 (0)	0 (0)

### Multifactor Logistic Regression Analysis of Factors Affecting SHAEs

To further investigate the pertinent factors influencing the occurrence of SHAEs, variables with *P*<.20 in Multimedia Appendix 1. were included in the multivariate logistic regression analysis, and the results (Table 3) showed that lymph node metastasis, Ccr of ≤60 mL per minute, RBC count of ≤3.3×10^12^ per liter, and combined therapy with vascular endothelial growth factor inhibitors (VEGFis) were risk factors influencing the occurrence of SHAEs (*P*<.05).

**Table 3:** Multivariable logistic regression analysis with backward elimination for predicting poly (adenosine diphosphate ribose) polymerase inhibitor (PARPi) serious hematological adverse events.

	*B*	OR^a^ (95% CI)	*P* value
Lymph node metastasis	1.907	6.733 (1.197-37.873)	.03
Ccr^b^≤60 mL per min	3.166	23.722 (3.121-180.303)	.002
RBC^c^ count≤3.3×10^12^ per liter	1.578	4.847 (1.020-23.041)	.047
PARPis combined with VEGFis^d^	1.909	6.749 (1.313-34.689)	.02

^a^OR: odds ratio.

^b^Ccr: creatinine clearance rate.

^c^RBC: red blood cell.

^d^VEGFi: vascular endothelial growth factor inhibitor.

### Development and Assessment of the Nomogram Model

**Figure 4:**
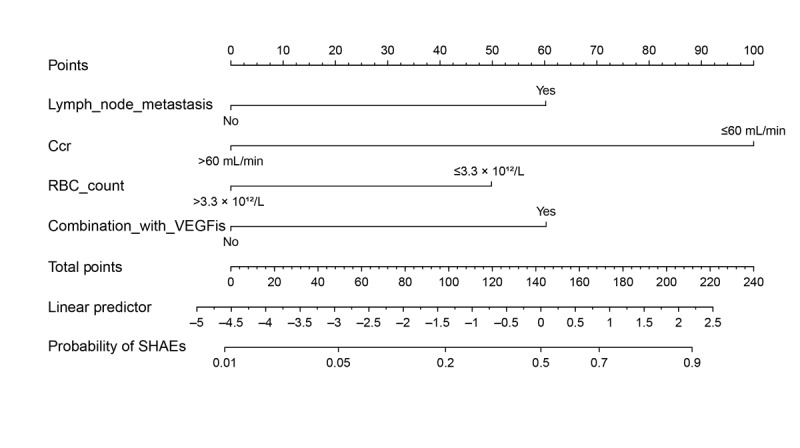
Nomogram predicting hematological adverse events (grade ≥3) in patients with ovarian cancer treated with poly (adenosine diphosphate ribose) polymerase inhibitors. Ccr: creatinine clearance rate; RBC: red blood cell; SHAE: serious hematological adverse event; VEGFi: vascular endothelial growth factor inhibitor.

**Figure 5:**
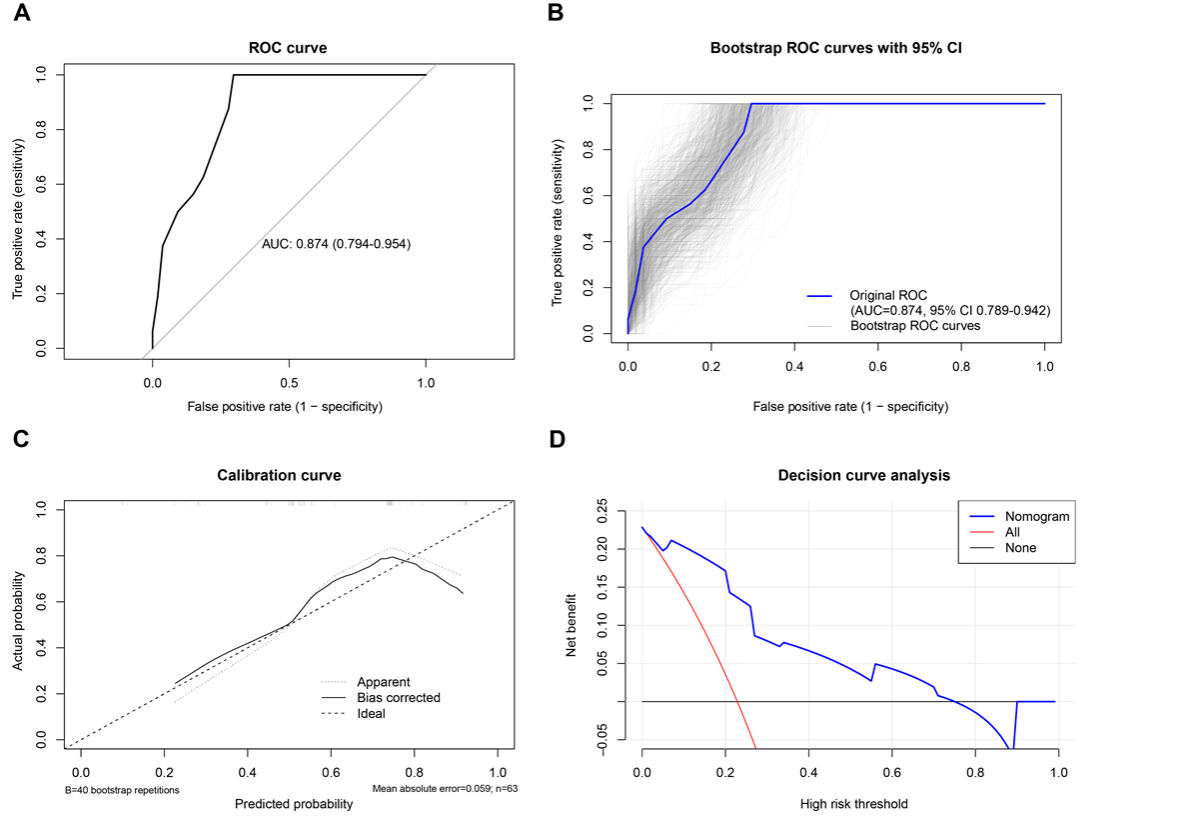
Calibration and clinical use of a diagnostic nomogram for predicting poly (adenosine diphosphate ribose) polymerase inhibitor serious hematological adverse events—(A) receiver operating characteristic (ROC) curve of the nomogram; (B) bootstrap ROC curve of the nomogram; (C) calibration curve of the nomogram; (D) decision curve analysis of the nomogram.

A nomogram model was developed using the R software to forecast SHAEs after receiving PARPi medication based on the findings of the multifactor analysis (Figure 4). The ROC curve was developed to evaluate this model’s predictive ability. With an AUC of 0.874, the nomogram model showed great predictive ability and consistency (Figure 5A). The ROC curve derived from bootstrap resampling maintained consistent stability in its positional trajectory (Figure 5B), suggesting that this phenomenon is more likely attributable to sample distribution and class imbalance issues common in small datasets rather than overfitting. The Hosmer-Lemeshow test was used to assess the model’s goodness of fit. According to the findings, the nomogram model developed in this study had a goodness of fit with a *P* value of .36. The model demonstrated moderate calibration (mean absolute error=0.061), with observed versus predicted risks aligning closely across deciles (Figure 5C). Decision curve analysis (Figure 5D) was conducted to evaluate the clinical utility of our risk prediction model. The curve demonstrates that the model yielded a positive net benefit at threshold probabilities ranging from 0% to 70%, suggesting that it can be safely applied to guide clinical interventions within this range. However, when the threshold probability exceeds 70%, the net benefit turns negative, indicating that the model-based decision strategy underperforms the “treat none” strategy (reference line net benefit=0) in this high-threshold range. This limitation may stem from either (1) an insufficient number of high-risk patients in the training dataset, leading to distributional bias and unreliable predictions in high-probability intervals; or (2) inherent inaccuracies in the model’s predictions for extreme-risk subgroups. Therefore, for patients within the high-threshold range (threshold probability>70%), additional confirmatory screening (eg, imaging examinations) or closer clinical monitoring should be prioritized to enhance decision accuracy rather than relying solely on this model to justify high-risk interventions.

## Discussion

### Principal Findings

During the treatment process with PARPis, most patients will experience varying degrees of adverse reactions, mainly mild or moderate, most commonly manifested as hematological adverse events, gastrointestinal adverse events, and fatigue. Most of the adverse events of a grade of ≥3 are hematological adverse events, which are the main reasons for adjusting drug dosage and interrupting and terminating medication, with a discontinuation rate of 27% [7-9]. SHAEs may also be associated with life-threatening declines in organ function and quality of life and even fatal outcomes. This study found that patients who experienced SHAEs showed a trend toward worse PFS than those without SHAEs (median 13.0, IQR 0.0-38.4 months vs median 24.0, IQR 19.2-28.8 months; *P*=.82); although no statistically significant difference was found, these toxicities still require early detection and proper management.

However, a previous study found that patients who experienced hematological adverse events while receiving PARPi treatment had much higher survival and response rates than those who did not experience toxicity. This finding may be connected to PARPis’ extensive cell dispersion [10]. Early adverse events related to PARPi hematological toxicity have been linked to antitumor activity, which may be a reliable and readily quantifiable clinical indicator in patients with ovarian cancer. In this study, although patients who developed hematological adverse events of a grade of ≥3 within the first month of PARPi treatment appeared to exhibit a higher DCR, this difference did not reach statistical significance. Conversely, the early efficacy of PARPis showed an inverse relationship with their associated hematological toxicities. Compared with patients who experienced SHAEs during treatment, patients who did not experience SHAEs showed higher DCR (94.4% vs 68.8%; *P*=.02) in the early stages of treatment (within 6 weeks), which may be due to the longer median time to onset of hematological adverse reactions of a grade of ≥3 reported in this study (median 41.0, IQR 29.3-52.7 months). Furthermore, it cannot be ruled out that the small-sample bias may have an impact on the research results. Although logistic regression analysis did not show that early efficacy could predict SHAEs related to PARPis, we still recommend closely monitoring relevant indicators for patients with poor early treatment response (within 6 weeks) to identify and intervene in adverse events early.

Of the 70 patients in this analysis, 16 (23%) experienced SHAEs, with a median time to onset of 41.0 months (IQR 29.3-52.7 months). In addition, 57% (40/70) experienced all-grade hematological adverse events, with the main onset time window falling between 7.4 and 26.6 months. The hematological adverse events of the patients in this study were mostly erythropenia and decreased hemoglobin regardless of severity, which was comparable to those found in previous research. Therefore, it is recommended that routine blood testing, especially RBC count and hemoglobin, be strengthened from the second year of patient medication. If necessary, preventive medication should be administered.

Lymph node metastasis was found to be an independent factor influencing the incidence of hematological adverse events using multivariate logistic regression analysis. Previous studies have demonstrated that patients with advanced solid tumors experience less tolerance to treatment. Lymph node status is not only a part of the International Federation of Gynecology and Obstetrics staging system for ovarian cancer but also an established prognostic factor in ovarian cancer [10-13]. A 13-year retrospective analysis suggests that, when it comes to patients with ovarian cancer, the lymph node ratio (calculated as the number of positive lymph nodes divided by the total number removed) is a more reliable prognostic indicator than the standard lymph node status [14]. As a result, PARPi users with lymph node metastasis in any pathological stage should be considered for further evaluation.

Previous studies have identified an association between PARPi dose and adverse events [9]. PARPis are predominantly eliminated via the kidneys. In clinical practice, creatinine and Ccr are used as markers to determine the glomerular filtration rate and functional parameters to assess renal function in patients. Patients with renal dysfunction are more likely to experience excessive accumulation of the drug or its active metabolites, which could result in severe adverse events. In general, low Ccr consistently appears to be a risk for drug adverse events, especially for drugs primarily excreted through the kidneys, and from the results of our study, this remains true for SHAEs related to PARPis. It is worth noting that elevated serum creatinine is a frequent adverse reaction to PARPis reported in previous studies [15-17], although most studies have shown that elevated serum creatinine may be caused by the inhibition of renal transporters but not accurately reflect a decline in glomerular filtration rate or renal failure [15,18]. Given the findings of this study, it is still advised that PARPi users with a Ccr of ≤60 mL per minute be routinely monitored.

This study found that an RBC count of ≤3.3×10^12^ per liter was a risk factor for SHAEs related to PARPis. The most plausible explanation for the association between erythrocyte reduction and hematological toxicity is impaired bone marrow reserve. Immature platelet fraction serves as an accurate hematological marker for assessing bone marrow function. However, as baseline immature platelet fraction testing is not routinely conducted in patients with ovarian cancer receiving PARPis at our institution, this study conducted a comprehensive evaluation of bone marrow reserve using indicators including RBC count, platelet count, hemoglobin, platelet-large cell ratio, mean corpuscular volume, and lactate dehydrogenase. Chi-square tests and multivariate logistic regression analysis revealed that none of the aforementioned parameters—except for RBC count—demonstrated a significant correlation with PARPi-related hematological adverse events. Insufficient evidence was found to support the hypothesis that SHAEs related to PARPis are primarily mediated by impaired bone marrow function. Instead, our findings may be better explained by the research by Molina et al [19] and Mehibel et al [20], which explores the association between the tumor microenvironment (TME) and the antitumor activity of PARPis. The RBCs are the efficient carriers of oxygen and other essential nutrients that are linked to aberrant metabolism in the TME, such as hypoxia. Hypoxia is a common characteristic of the TME in solid tumors and is caused by the rapid growth and proliferation of malignant solid tumors and the underdevelopment of vascular system tissues, resulting in local hypoxic tension in tissues; hypoxia, in turn, regulates the angiogenesis and growth of tumor tissue, promoting tumor invasion and metastasis, to adapt to hypoxic and nutrient-limited environments [19,21]. This causal transformation results in most solid tumors being in a state of moderate (1%-2% oxygen) to severe (<0.01% oxygen) hypoxia internally [22]. In the female population, the normal RBC count is 4.0 to 5.0×10^12^ per liter. When it is below the lower limit of the normal range, the ability of blood to transport oxygen is weakened, which may further exacerbate the hypoxic condition of the TME. It has been found that hypoxia can induce the overexpression of breast cancer resistance protein and multiple drug resistance protein [23] and the activation of the transforming growth factor beta, transforming growth factor alpha and epidermal growth factor receptor, and cyclooxygenase-2 pathways [24-26] by regulating the hypoxia-inducible factor α and cancer-associated fibroblasts, thus inducing therapeutic resistance. The study by Mehibel et al [20] revealed that cancer cells exhibited increased resistance to PARPis under moderate hypoxia (2% oxygen) when compared to their normoxic counterparts, suggesting a hypoxia-induced protective mechanism. However, under severe hypoxia (0.5% oxygen), cancer cells showed the same significant increase in sensitivity to PARPis regardless of whether HRRD was negative or positive. Notably, the interpretation of our results based on those of these previous studies remains limited by the sample size, cross-sectional design, and suboptimal sensitivity of bone marrow evaluation methods, necessitating future validation in prospective cohorts with dynamic bone marrow function monitoring.

In addition, this study found that PARPi combination with VEGFis was one of the risk factors for SHAEs during PARPi treatment. The VEGFis used in patients undergoing combination therapy in this study included bevacizumab, apatinib, and lenvatinib. Previous literature has reported a significant proportion of hematological adverse events of a grade of ≥3 during the use of VEGFi monotherapy [27-29]. Notably, neutropenia and thrombocytopenia have been reported to occur more frequently with VEGFis monotherapy than with PARPis monotherapy, although the proportion of grade ≥ 3 anemia accounts for less than 4% of cases. The hematological toxicity of VEGFis may arise from their suppression of vascular endothelial growth factor (VEGF) signaling, which disrupts the bone marrow microenvironment, leading to vascular regression, hypoxia, and impaired stromal cell function, thereby compromising hematopoietic stem cell survival and differentiation. Concurrently, blockade of VEGF receptors (eg, VEGF receptors 1 and 2) on hematopoietic progenitor cells can interfere with erythroid, myeloid, and megakaryocytic maturation, resulting in anemia, neutropenia, and thrombocytopenia. Furthermore, VEGF inhibition may upregulate proinflammatory cytokines (eg, tumor necrosis factor and interleukin-6) and hepcidin, further suppressing hematopoiesis or inducing iron-restricted anemia. These mechanisms collectively contribute to the potential hematological adverse effects associated with VEGFis [30]. PARPis cause anemia by binding to poly (adenosine diphosphate ribose) polymerase 2 in the RBCs, leading to shortened life span and increased absorption of RBCs [31]. The possible mechanism of PARPi-mediated bone marrow suppression also includes inhibiting poly (adenosine diphosphate ribose) polymerase 1 in hematopoietic stem cells, interfering with neutrophil, platelet, and RBC formation [32]. Thus, it is hypothesized that the 2 together could worsen overall hematological toxicity via distinct mechanisms, increasing the risk of hematological adverse events of a grade of ≥3 in patients. This phenomenon can also be observed in the PAOLA-1 (PArp inhibitor OLaparib in Advanced ovarian cancer 1) clinical study of olaparib combined with bevacizumab in ovarian cancer treatment [33].

Other than the previously discussed factors, further parameters may modulate the development of SHAEs from PARPi therapy. Previous studies have found that *BRCA1* and *BRCA2* mutation or HRRD positivity is a risk factor for hematological adverse events related to PARPis [34-36]. Moreover, the Spanish Society of Medical Oncology clinical guidelines [37] for ovarian cancer indicate that PARPis induce cell death in HRRD-positive tumor cells through a “synthetic lethality” mechanism, with *BRCA1* and *BRCA2* mutations being one of the established causes of HRRD in tumors. Therefore, the guidelines recommend *BRCA* mutation testing in patients before PARPi therapy. However, due to the high cost of testing, this study found that the willingness of most patients with cancer to undergo *BRCA* and HRRD testing was low, with a detection rate of less than 55%. Therefore, it is difficult to verify the impact of *BRCA* and HRRD status on PARPi SHAEs. A network meta-analysis indicated that there was a significant difference in the risk of any adverse events of a grade of ≥3 across PARPi treatments, and based on the surface under the cumulative ranking curve rankings, olaparib was the safest PARPi in terms of risk of hematological adverse events [6]. Some studies have also found that hypertension, low baseline hemoglobin level, and low baseline hematocrit level are related to the occurrence of hematological adverse events induced by PARPi monotherapy [35]. Due to data limitations in this study, definitive comparative analyses could not be conducted, warranting future studies to address this gap.

On the basis of the identified predictive factors, this study developed a nomogram model to provide a user-friendly method for early prediction of SHAEs associated with PARPis in clinical practice. Internal validation demonstrated that this risk assessment model exhibited moderate accuracy and predictive capability, with an ROC curve AUC of 0.874 and Hosmer-Lemeshow test of *P*=.36. These findings provide evidence for developing personalized treatment strategies in clinical practice and suggest potential clinical utility.

This study also has several limitations. First, although repeated cross-validation showed no strong signs of overfitting, the small sample size limited our ability to fully address associated challenges such as data distribution biases and class imbalance. Furthermore, external validation, which is essential for confirming generalizability, could not be conducted due to the unavailability of an independent external cohort. This is a common constraint in single-center studies of rare conditions. Future multi-institutional studies are warranted to externally validate our findings. The analysis was further limited by missing data for several key prognostic indicators (eg, *BRCA* and HRRD status), which precluded their inclusion in the model. Finally, as the model was developed and validated exclusively on a Chinese population, its generalizability to other ethnic groups requires further investigation.

### Conclusions

This study used objective data widely used in clinical settings to create and internally test a basic predictive model of SHAEs for PARPi users with ovarian cancer. The clinical nomogram included the 4 best predictors of PARPi SHAEs: lymph node metastasis, Ccr, RBC count, and therapeutic schedule. This should help identify patients who need closer monitoring for SHAEs and more rigorous treatment.

## Data Availability

The datasets generated or analyzed during this study are available from the corresponding author on reasonable request.

## Appendix

Multimedia Appendix 1 Comparison of clinical characteristics between the 2 groups (N=70).

## Abbreviations

AUC: area under the curve
Ccr: creatinine clearance rate
DCR: disease control rate
HRRD: homologous recombination repair deficiency
OR: odds ratio
PAOLA-1: PArp inhibitor OLaparib in Advanced ovarian cancer 1
PARPi: poly (adenosine diphosphate ribose) polymerase inhibitor
PFS: progression-free survival
RBC: red blood cell
ROC: receiver operating characteristic
SHAE: serious hematological adverse event
TME: tumor microenvironment
VEGF: vascular endothelial growth factor
VEGFi: vascular endothelial growth factor inhibitor
