# Supplementary material for: Model for Predicting Serious Hematological Adverse Events in Individuals With Ovarian Cancer Receiving Poly (Adenosine Diphosphate Ribose) Polymerase Inhibitor Treatment: Prospective Cohort Study
Source: JMIR Med Inform. 2025 Nov 12;13:e72994. doi: 10.2196/72994 (PMC12658389; doi:10.2196/72994)
Supplement: Multimedia Appendix 1 [file medinform_v13i1e72994_app1.docx]

**Table 1.** Comparison of clinical characteristics between the 2 groups (N=70).

| Clinical characteristic | | Total, n (%) | SHAE^a^ group, n (%) | | *P* value |
| --- | --- | --- | --- | --- | --- |
|  | |  | No SHAEs (n=54) | SHAEs (n=16) |  |
|  | | | | | |
| **Age (y)** | | | | | .73 |
|  | <65 | 57 (81) | 43 (80) | 14 (88) |  |
|  | ≥65 | 13 (19) | 11 (20) | 2 (12) |  |
| **Ethnicity** | | | | | .37 |
|  | Han nationality | 46 (66) | 34 (63) | 12 (75) |  |
|  | Zhuang nationality | 24 (34) | 20 (37) | 4 (25) |  |
| **Marital status** | | | | | .23 |
|  | Married | 69 (99) | 54 (100) | 15 (94) |  |
|  | Unmarried | 1 (1) | 0 (0) | 1 (6) |  |
| **Menopause status** | | | | | >.99 |
|  | Postmenopausal | 68 (97) | 52 (96) | 16 (100) |  |
|  | Premenopausal | 2 (3) | 2 (4) | 0 (0) |  |
| **BMI (kg/m^2^)** | | | | | >.99 |
|  | <25.0 | 53 (76) | 41 (76) | 12 (75) |  |
|  | ≥25.0 | 17 (24) | 13 (24) | 4 (25) |  |
| **Hypertension** | | | | | >.99 |
|  | Yes | 10 (14) | 8 (15) | 2 (12) |  |
|  | No | 60 (86) | 46 (85) | 14 (88) |  |
| **Family history of cancer** | | | | | >.99 |
|  | Yes | 17 (24) | 13 (24) | 4 (25) |  |
|  | No | 53 (76) | 41 (76) | 12 (75) |  |
| **Previous debulking surgery therapy** | | | | | —^b^ |
|  | Yes | 70 (100) | 54 (100) | 16 (100) |  |
|  | No | 0 (0) | 0 (0) | 0 (0) |  |
| **Previous radiotherapy** | | | | | .41 |
|  | Yes | 2 (3) | 1 (2) | 1 (6) |  |
|  | No | 68 (97) | 53 (98) | 15 (94) |  |
| **Gravidity** | | | | | .13 |
|  | <3 | 32 (46) | 22 (41) | 10 (62) |  |
|  | ≥3 | 38 (54) | 32 (59) | 6 (38) |  |
| **Number of artificial abortions** | | | | | .42 |
|  | <2 | 49 (70) | 36 (67) | 13 (81) |  |
|  | ≥2 | 21 (30) | 18 (33) | 3 (19) |  |
| **History of induced abortions** | | | | | >.99 |
|  | Yes | 1 (1) | 1 (2) | 0 (0) |  |
|  | No | 69 (99) | 53 (98) | 16 (100) |  |
| **ECOG^c^ score** | | | | | >.99 |
|  | <1 | 68 (97) | 52 (96) | 16 (100) |  |
|  | ≥1 | 2 (3) | 2 (4) | 0 (0) |  |
| **Subtype of ovarian carcinoma** | | | | | .99 |
|  | High-grade serous carcinoma | 59 (84) | 45 (83) | 14 (88) |  |
|  | Other | 11 (16) | 9 (17) | 2 (12) |  |
| **FIGO^d^ stage** | | | | | .71 |
|  | <3 | 9 (13) | 6 (11) | 3 (19) |  |
|  | ≥3 | 61 (87) | 48 (89) | 13 (81) |  |
| **Maximum tumor diameter (mm)** | | | | | >.99 |
|  | <25 | 20 (29) | 15 (28) | 5 (31) |  |
|  | ≥25 | 50 (71) | 39 (72) | 11 (69) |  |
| **Lymph node metastasis** | | | | | .10 |
|  | Yes | 31 (44) | 21 (39) | 10 (62) |  |
|  | No | 39 (56) | 33 (61) | 6 (38) |  |
| **Distant metastasis** | | | | | .55 |
|  | Yes | 67 (96) | 52 (96) | 15 (94) |  |
|  | No | 3 (4) | 2 (4) | 1 (6) |  |
| **Number of distant metastases** | | | | | .92 |
|  | <2 | 16 (23) | 13 (24) | 3 (19) |  |
|  | ≥2 | 54 (77) | 41 (76) | 13 (81) |  |
| **Degree of differentiation** | | | | | .43 |
|  | Moderately and poorly differentiated | 60 (86) | 46 (85) | 14 (88) |  |
|  | Well differentiated | 2 (3) | 1 (2) | 1 (6) |  |
|  | Unknown^b^ | 8 (11) | 7 (13) | 1 (6) |  |
| **Breast cancer gene status** | | | | | >.99 |
|  | Positive | 21 (30) | 18 (33) | 3 (19) |  |
|  | Negative | 17 (24) | 15 (28) | 2 (12) |  |
|  | Unknown^b^ | 32 (46) | 21 (39) | 11 (69) |  |
| **HRRD^e^ status** | | | | | —^b^ |
|  | Positive | 10 (14) | 10 (19) | 0 (0) |  |
|  | Negative | 4 (6) | 2 (4) | 2 (12) |  |
|  | Unknown^b^ | 56 (80) | 42 (78) | 14 (88) |  |
| **Duration of the disease (y)** | | | | | .66 |
|  | <2 | 40 (57) | 30 (56) | 10 (62) |  |
|  | ≥2 | 30 (43) | 24 (44) | 6 (38) |  |
| **Treatment line** | | | | | .13 |
|  | First line | 67 (96) | 53 (98) | 14 (88) |  |
|  | Second line and beyond | 3 (4) | 1 (2) | 2 (12) |  |
| **PFI^f^ (mo)** | | | | | .19 |
|  | <24 | 54 (77) | 27 (50) | 11 (69) |  |
|  | ≥24 | 16 (23) | 27 (50) | 5 (31) |  |
| **Ccr^g^ (mL per min)** | | | | | .005 |
|  | ≤60 | 12 (17) | 5 (9) | 7 (44) |  |
|  | >60 | 58 (83) | 49 (91) | 9 (56) |  |
| **Hematocrit level** | | | | | .43 |
|  | ≤0.35 | 54 (77) | 40 (74) | 14 (88) |  |
|  | >0.35 | 16 (23) | 14 (26) | 2 (12) |  |
| **RBC^h^ count** | | | | | .01 |
|  | ≤3.3 × 10^12^ per liter | 37 (53) | 24 (44) | 13 (81) |  |
|  | >3.3 × 10^12^ per liter | 33 (47) | 30 (56) | 3 (19) |  |
| **Platelet count** | | | | | .28 |
|  | <125 × 10^9^ per liter | 53 (76) | 43 (80) | 10 (62) |  |
|  | ≥125 × 10^9^ per liter | 17 (24) | 11 (20) | 6 (38) |  |
| **Hb^i^ level (g/L)** | | | | | >.99 |
|  | ≤116 | 60 (86) | 46 (85) | 14 (88) |  |
|  | >116 | 10 (14) | 8 (15) | 2 (12) |  |
| **Platelet-large cell ratio (%)** | | | | | .77 |
|  | <13 | 8 (11) | 7 (13) | 1 (6) |  |
|  | ≥13 | 62 (89) | 47 (87) | 15 (94) |  |
| **Mean corpuscular volume (fL)** | | | | | .48 |
|  | 86-100 | 36 (51) | 29 (54) | 7 (44) |  |
|  | <86 or >100 | 34 (49) | 25 (46) | 9 (56) |  |
| **Lactate dehydrogenase (U/L)** | | | | | .30 |
|  | ≤227 | 63 (90) | 47 (87) | 16 (100) |  |
|  | >227 | 7 (10) | 7 (13) | 0 (0) |  |
| **CA125^j^ (kU/L)** | | | | | .87 |
|  | <30.0 | 56 (80) | 45 (83) | 11 (69) |  |
|  | ≥30.0 | 11 (16) | 8 (15) | 3 (19) |  |
|  | Unknown^b^ | 3 (4) | 2 (4) | 1 (6) |  |
| **HE4^k^ (pmol/L)** | | | | | .42 |
|  | <80 | 48 (69) | 39 (72) | 9 (56) |  |
|  | ≥80 | 19 (27) | 13 (24) | 6 (38) |  |
|  | Unknown^b^ | 3 (4) | 2 (4) | 1 (6) |  |
| **Ki-67^l^ level** | | | | | .36 |
|  | <0.75 | 25 (36) | 21 (39) | 4 (25) |  |
|  | ≥0.75 | 11 (16) | 7 (13) | 4 (25) |  |
|  | Unknown^b^ | 34 (49) | 26 (48) | 8 (50) |  |
| **SII^m^** | | | | | .22 |
|  | <500 | 30 (43) | 21 (39) | 9 (56) |  |
|  | ≥500 | 40 (57) | 33 (61) | 7 (44) |  |
| **NLR^n^** | | | | | 72 |
|  | <4.50 | 41 (59) | 31 (57) | 10 (62) |  |
|  | ≥4.50 | 29 (41) | 23 (43) | 6 (38) |  |
| **PLR^o^** | | | | | .59 |
|  | <230 | 51 (73) | 38 (70) | 13 (81) |  |
|  | ≥230 | 19 (27) | 16 (30) | 3 (19) |  |
| **Previous adverse events on chemotherapy** | | | | | .73 |
|  | Yes | 57 (81) | 43 (80) | 14 (88) |  |
|  | No | 13 (19) | 11 (20) | 2 (12) |  |
| **Type of PARPi^p^** | | | | | .66 |
|  | Fluzoparib | 5 (7) | 5 (9) | 0 (0) |  |
|  | Olaparib | 46 (66) | 35 (65) | 11 (69) |  |
|  | Niraparib | 19 (27) | 14 (26) | 5 (31) |  |
| **PARPis combined with VEGFis^q^** | | | | | .10 |
|  | Yes | 14 (20) | 8 (15) | 6 (38) |  |
|  | No | 56 (80) | 46 (85) | 10 (62) |  |
| **Response to 6-wk treatment** | | | | | .02 |
|  | CR^r^, PR^s^, or SD^t^ | 62 (89) | 51 (94) | 11 (69) |  |
|  | PD^u^ | 8 (11) | 3 (6) | 5 (31) |  |

^a^SHAE: serious hematological adverse event.

^b^The chi-square test was conducted for comparisons of categorical variables using valid cases only (excluding missing data). The homologous recombination repair deficiency status variable had a high proportion of missing data (56/70, 80% of cases) and, therefore, was excluded from statistical comparison; a *P* value is not applicable and is indicated by an em dash.

^c^ECOG: Eastern Cooperative Oncology Group performance status.

^d^FIGO: International Federation of Gynecology and Obstetrics.

^e^HRRD: homologous recombination repair deficiency.

^f^PFI: platinum-free interval.

^g^Ccr: creatinine clearance rate.

^h^RBC: red blood cell.

^i^Hb: hemoglobin.

^j^CA125: carbohydrate antigen 125.

^k^HE4: human epididymis protein 4.

^l^Ki-67: marker of proliferation Kiel 67.

^m^SII: systemic immune inflammation index (platelet count × [neutrophil count/lymphocyte count]).

^n^NLR: neutrophil-to-lymphocyte ratio (neutrophil count/lymphocyte count).

^o^PLR: platelet-to-lymphocyte ratio (platelet count/lymphocyte count).

^p^PARPi: poly (adenosine diphosphate ribose) polymerase inhibitor.

^q^VEGFi: vascular endothelial growth factor inhibitor.

^r^CR: complete response.

^s^PR: partial response.

^t^SD: stable disease.

^u^PD: progressive disease.
